# Bird brains: Does absolute size matter?

**DOI:** 10.3758/s13420-016-0247-9

**Published:** 2016-09-23

**Authors:** Jackie Chappell

**Affiliations:** School of Biosciences, University of Birmingham, Edgbaston, Birmingham B15 2TT UK

**Keywords:** Cognitive ethology, Comparative cognition, Brain size, Birds, Self-regulation

## Abstract

Kabadayi, Taylor, von Bayern, and Osvath (2016, *Royal Society Open Science*, *3*, 160104) recently showed that among birds, absolute brain size predicts performance on a motor self-control task thought to be important for cognition. However, birds performed at an equivalent level to much larger-brained primates, opening up the debate about brain size and cognition.

We humans are proud of our intelligence (as shown by the species name we gave ourselves: *sapiens*), so it is understandable that we are deeply interested in the evolution of complex, flexible cognition in other species. Furthermore, since the brain is the organ of cognition, there has been a long and sometimes controversial tradition of trying to predict intelligence on the basis of brain size, from the assumption that a bigger brain must be a better brain. This line of research has been bedeviled by two key problems: What is the best way to measure brain size, and how can cognitive abilities be measured and compared between species? Historically, absolute brain size (measured by mass or volume) was used, until it was noted that there is an underlying allometric relationship between brain size and body size. This led to the use of relative measures of brain size that take into account the known scaling relationships between brain size and body size for particular taxonomic groups, such as mammals. However, more recently evidence has emerged that absolute brain size predicts key cognitive abilities such as self-regulation (e.g., MacLean et al., 2014). This, in turn, raises a thorny problem: some taxa of birds (notably crows and parrots) perform at a similar level to primates on tests of cognition, and yet their brains are tiny in comparison to those of primates.

Kabadayi, Taylor, von Bayern, and Osvath (2016) set out to investigate the relationship between brain volume and self-regulation in birds, extending and improving upon work by MacLean and colleagues (2014), which had focused primarily on mammals, and more specifically on primates (23 of the 36 species tested). As was mentioned above, selecting an appropriate measure of cognitive ability is fraught with difficulty and controversy. However, one important building block of flexible cognition is the ability to inhibit or withhold responses, which enables the individual to avoid falling into “tempting traps” when withholding a response would provide a better reward or result in greater efficiency. Although this is only one component of cognition, measuring species’ self-regulatory abilities ought to provide a useful, basic assay of cognition relative to brain size. Kabadayi et al. replicated the single task from MacLean et al. (2014) that they considered most clearly related to motor self-regulation: the cylinder task. In this task, subjects were confronted with an opaque cylinder with open ends, which had food at its center. Over a series of familiarization trials, subjects reached into the tube through either open end to retrieve the food, and—once familiar with this process—were then tested with an identical, but now transparent, tube. The “tempting trap” was that they might now to try to reach directly for the food through the walls of the cylinder, rather than accessing the tube from the ends, as before. Falling into this tempting trap is considered a failure of self-regulation.

Kabadayi et al. (2016) tested three corvid species (ravens, New Caledonian crows, and jackdaws) on the cylinder task, pooling their data with trial-level data obtained from the seven bird species tested on the same task by MacLean et al. (2014). They then constructed two models to test the effects of absolute and residual brain volume on performance in the cylinder task, where residual brain volume was calculated from the predicted relationship between absolute brain volume and body size, correcting for phylogenetic relatedness. The researchers found that both measures of brain volume significantly predicted performance, but that absolute brain size was a stronger predictor than residual brain size.

However, although the data clearly supported relationships between both absolute and relative brain size and performance within the taxonomic group tested, the key finding from this study was that the crows’ performance equaled or exceeded that of the primates tested by MacLean et al. (2014), despite the absolute volume of the crows’ brains being much smaller. For example, ravens’ performance equaled that of chimpanzees, despite the volume of chimpanzees’ brains being more than 25 times that of ravens. Similarly, jackdaws’ performance outranked that of capuchin monkeys, despite a more than 12-fold difference in brain volume. Thus, whereas absolute brain size clearly influenced performance on this task *within* a taxonomic group, it cannot explain differences *between* taxonomic groups.

In their discussion of this paradox, Kabadayi et al. (2016) suggested that the solution may lie in comparing the numbers of neurons rather than brain size, or in comparing the sizes of particular brain regions between species. Excitingly, both predictions have been supported by a more recent study by Olkowicz and colleagues (2016), who used the technique of isotropic fractionation to count the numbers of neurons in the brains of various species of parrot and song bird (including corvids) and compared this to previously published data on mammals. They found that birds’ brains contained many more neurons than mammalian (and even primate) brains of a similar mass. In fact, the brains of songbirds and parrots contained about twice as many neurons as those of similarly sized primates. Furthermore, the birds pack proportionally more of their neurons in forebrain regions (such as the telencephalon) than do mammals, and this proportion increases with brain size. It seems that the weight constraint imposed upon birds by the demands of flight has resulted in the evolution of lightweight but high-performance brains. Each neuron is smaller than those of other taxa, but densely packed in the brain, with proportionally more in the telencephalon, all of which may facilitate interneuronal communication and result in higher processing speeds in avian brains.

These are exciting advances that may allow us to resolve the tangle of conflicting evidence about the relationship between brain size and cognition that has previously characterized the field. However, substantial gaps in our knowledge remain. The study by Kabadayi et al. (2016) used a single measure of self-regulation as a proxy for cognition, but cognition is undoubtedly a complex and multifaceted suite of abilities. A more diverse range of tasks resulting in a convergent pattern of results would improve confidence in the result, and also mitigate potential problems with the task that might influence performance but that do not indicate differences in the ability being tested. For example, four of the bird species tested (though none of the three corvid species) improved their performance significantly across the testing trials, suggesting that experience with the features of the experimental apparatus (particularly transparency, as the authors themselves noted) may be important in influencing performance.

Designing a range of cognitive tests will require us to understand the elements that make up flexible, complex cognition in much greater detail. Although species undoubtedly share similar underlying cognitive processes, the ways in which these are expressed are likely to vary substantially, depending on the species’ morphology, ontogeny, life history, social system, and ecological niche. Specifically, what do birds (or primates, or cetaceans, for that matter) actually *do* with all this brain power in the wild? Brains are metabolically expensive organs, so presumably the cognitive power that a large brain delivers must provide a correspondingly substantial fitness advantage. The requirements imposed by complex social systems and varied diets have been suggested as selection pressures promoting complex cognition. However, the evidence for such a relationship is patchy, and strongest within primates: It does not hold more generally across other taxa, such as birds, in which other advantages of large brains, such as the ability to adapt to novel environmental conditions, have been suggested (e.g., Sol, Duncan, Blackburn, Cassey, & Lefebvre, 2005). Tightly controlled laboratory experiments on captive birds are vital for probing the details of fundamental mechanisms, but we need to combine these with observations and “field experiments” on wild bird species in order to determine why birds need such efficient but high-performance brains, and what they use them for.

## References

[CR1] Kabadayi C, Taylor LA, von Bayern AMP, Osvath M (2016). Ravens, New Caledonian crows and jackdaws parallel great apes in motor self-regulation despite smaller brains. Royal Society Open Science.

[CR2] MacLean EL, Hare B, Nunn CL, Addessi E, Amici F, Anderson RC, Zhao Y (2014). The evolution of self-control. Proceedings of the National Academy of Sciences.

[CR3] Olkowicz S, Kocourek M, Lučan RK, Porteš M, Fitch WT, Herculano-Houzel S, Němec P (2016). Birds have primate-like numbers of neurons in the forebrain. Proceedings of the National Academy of Sciences.

[CR4] Sol D, Duncan RP, Blackburn TM, Cassey P, Lefebvre L (2005). Big brains, enhanced cognition, and response of birds to novel environments. Proceedings of the National Academy of Sciences.

